# Single-center experience with mesh suture for primary parastomal hernia repair

**DOI:** 10.1007/s00464-026-13118-4

**Published:** 2026-07-17

**Authors:** Joe Banton, Jeffrey A. Blatnik, Robert MacGregor, Arnab Majumder

**Affiliations:** Section of Minimally Invasive Surgery, Department of Surgery, Washington University School of Medicine, 660 S Euclid Ave, Box 8109, St. Louis, MO 63105 USA

**Keywords:** Parastomal hernia repair, Mesh suture, Recurrent parastomal hernia, Primary parastomla hernia repair

## Abstract

**Introduction:**

Parastomal hernias remain a persistent challenge for hernia surgeons due to significant wound morbidity and high rates of hernia recurrence. Enlarging of the stomal aperture due to suture pull-through or de novo fascial separation can lead to recurrence despite repair technique. Mesh suture (Duramesh, Mesh Suture Inc, Chicago, IL) is designed to distribute tension across multiple filaments, theoretically reducing pull-through. We analyzed our outcomes of the use of Duramesh as an alternative to primary suture closure in the repair of primary and recurrent parastomal hernias.

**Methods:**

We conducted a single-center, retrospective review of a prospectively maintained series of patients who underwent primary repair of parastomal hernias using Duramesh between January 2024 and July 2025. We included both primary and recurrent parastomal hernias with no exclusion based on prior repair type or defect size.

**Results:**

Forty-two patients were included. Mean age was 66 ± 10.9 years, mean BMI was 31 kg/m^2^, and 54.8% were male. Stoma types included colostomy (47.6%), ileostomy (28.6%), and urostomy (21.4%). Fifty-four percent had undergone at least one prior PSH repair. Median hernia defect size was 20 cm^2^ (range 2–120 cm^2^). Mean operative time was 225 min and mean length of stay was 6.4 days. Surgical site occurrences were identified in 26.2% of patients, including a 7.1% SSI rate. At a mean follow-up of 7.1 months, 54.8% of patients developed hernia recurrence, with a mean time to recurrence of 6.4 months. Patients undergoing redo parastomal hernia repair had significantly higher recurrence rates compared to those undergoing first time repair (61.5% vs 30.8%, *p* = 0.050).

**Conclusions:**

Primary PSH repair with Duramesh alone is associated with high recurrence rates (54.8%) and should not be considered a durable standalone strategy. Though suture pull-through was not identified as a failure mechanism in this limited series, the persistent stomal aperture renders primary repair insufficient regardless of suture construct.

**Graphical abstract:**

**Figure:**
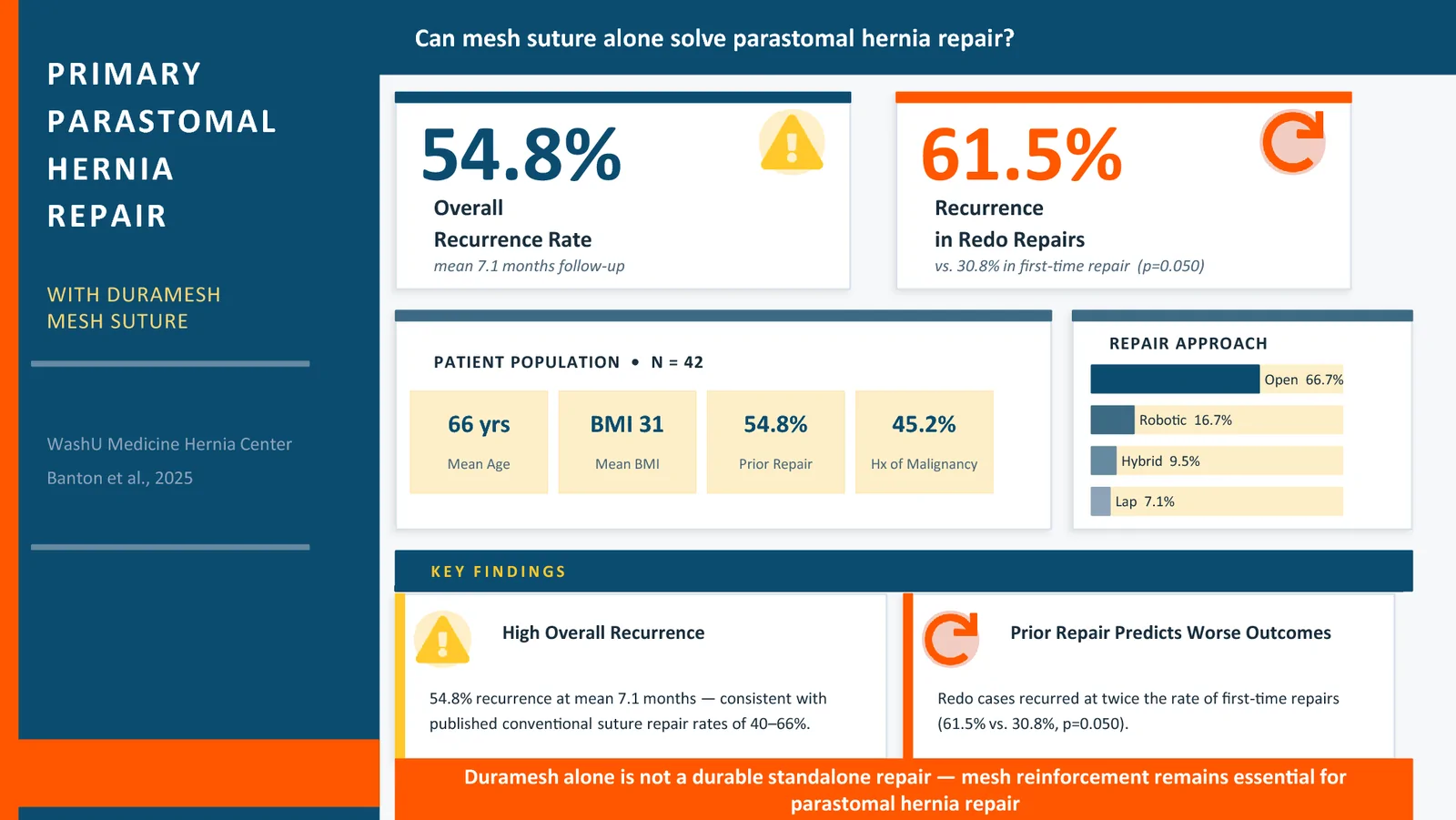

Intestinal stoma creation is a common surgical procedure, with an estimated 100,000 to 120,000 new ostomies created annually in the United States [1]. Parastomal hernia (PSH) is the most frequent long-term complication of stoma formation with the reported prevalence varying from 30 to 60% [2, 3]. Parastomal hernias may arise within the first two years following stoma creation, though the risk of herniation increases over time and is often viewed as an inevitable sequela of having a permanent stoma [4].

Parastomal hernias impose a substantial burden to both patients and the healthcare system. Patients with parastomal hernias frequently experience pain, difficulties with stoma appliance fitting and leakage, peristomal skin complications, and deterioration in quality of life [5, 6]. Beyond the lifestyle factors affecting patients on a day-to-day basis, hernia formation may lead to serious complications including bowel obstruction, incarceration, and strangulation, sometimes necessitating emergent operative intervention. Nationwide registry data demonstrate that annual rates of parastomal hernia repair have more than doubled over recent decades, reflecting the large population of patients living with permanent stomas and the persistent challenge of providing a durable repair [7]. Despite this growing surgical burden, long-term outcomes following repair remain poor, with previous studies identifying a 5-year cumulative reoperation rate exceeding 20%, and long-term registry studies demonstrating recurrence rates approaching 40% at 15 years—the highest of any abdominal wall hernia subtype [8, 9]. This is in direct opposition to the trends seen for incisional hernia repair with long-term rates of recurrence dropping below 10% in patients who undergo wide sublay based mesh repairs.

Given the high risk of recurrence in primary suture repairs, mesh-based repairs have become the gold standard approach in the repair of parastomal hernias [10, 11]. The most frequently used mesh-based techniques are the keyhole and Sugarbaker approaches, both of which employ prosthetic mesh in an intraperitoneal onlay or a retromuscular/preperitoneal sublay position. The keyhole technique requires a slit or cruciate incision in the mesh that allows the conduit to pass through, providing adequate mesh overlap along the abdominal wall defect. With the Sugarbaker technique, the stoma is lateralized beneath the mesh to cover both the stoma aperture and conduit.

Despite the evidence favoring mesh-based approaches, there remain clinical scenarios in which these mesh repair options are not feasible or prudent, including in contaminated fields or in patients with high burden of intra-abdominal adhesions. In these settings, primary suture repair is often utilized despite high rates of recurrence, which has been shown most often to be due to suture pull-through and fascial disruption at the suture-tissue interface. Tension at the suture-tissue interface is the most common failure point of most primary suture repair as the concentrated force applied by traditional suture can cut through the fascia and muscle, which can be particularly problematic in attenuated or retracted fascia [12].

Duramesh (Mesh Suture Inc., Chicago, IL) is a novel, FDA-approved suture that attempts to address this fundamental limitation. By distributing tension across multiple filaments along the length of the suture, Duramesh works to reduce peak stress at the suture-tissue interface, thereby mitigating suture pull-through [12]. Initial registry data, single-center series, and early systematic reviews have demonstrated acceptable short-term surgical site occurrence and recurrence rates following laparotomy closure and ventral hernia repair [13–15].

Despite the theoretical rationale for its application in parastomal hernia repair, there are no dedicated series examining the use of Duramesh specifically for primary PSH repair across a heterogeneous population of colostomy, ileostomy, and urostomy patients. The purpose of this study is to evaluate a single-center experience utilizing mesh suture for primary parastomal hernia repair, with a focus on perioperative outcomes, surgical site morbidity, and short-term hernia recurrence.

## Methods

The study was approved by the Washington University Institutional Review Board (IRB). A retrospective review was performed to identify all patients who underwent primary repair of a parastomal hernia using mesh suture (Duramesh, Mesh Suture Inc., Chicago, IL) between January 2024 and July 2025 by surgeons at a tertiary academic center.

Data reviewed and collected included patient demographic information such as age, sex, body mass index (BMI), race, smoking status, and comorbidities. Ostomy-specific data were recorded including stoma type (colostomy, ileostomy, or urostomy), ostomy configuration (end vs. loop), and ostomy location. History of prior parastomal hernia repair was documented, including the type of prior repairs (primary suture, Sugarbaker, or keyhole). Hernia characteristics were recorded along with operative details including repair technique and operative time. Postoperative outcomes included length of hospital stay, surgical site occurrences (SSO), surgical site infections (SSI), postoperative ileus, hernia recurrence, time to recurrence, and duration of follow-up.

All statistical analyses were performed using Microsoft Excel (Microsoft Corporation, Redmond, WA). Categorical variables are presented as frequencies and percentages. Continuous variables are presented as means with standard deviation or medians with range, as appropriate based on distribution. For all comparisons, a *p* value < 0.05 was considered statistically significant.

## Results

A total of 42 patients who underwent primary repair of a parastomal hernia with Duramesh between January 2024 and July 2025 were identified and included in the analysis.

### Patient demographics and clinical characteristics

Baseline demographic and clinical characteristics are summarized in Table 1. The cohort consisted of 23 male patients (54.8%) and 19 female patients (45.2%). The mean age at time of parastomal hernia repair was 66 ± 10.9 years (range 36–82). The mean body mass index (BMI) at time of repair was 31 ± 6.9 kg/m^2^.

**Table 1:** Baseline patient demographics and clinical characteristics (*N* = 42)

Characteristic	*N*	%
*Total patients*	42	–
Age, mean ± SD (range), years	66 ± 10.9 (36–82)	–
BMI, mean ± SD, kg/m^2^	31 ± 6.9	–
*Sex*		
Male	23	54.8%
Female	19	45.2%
*Race*		
White	41	97.6%
Other	1	2.4%
*Smoking status*		
Never	24	57.1%
Former	12	28.6%
Current	6	14.3%
Prior radiation	12	28.6%
Active chemotherapy at time of repair	1	2.4%
History of active or prior malignancy	19	45.2%
*Comorbidities*		
Hypertension	31	73.8%
Hyperlipidemia	20	47.6%
Diabetes mellitus	15	35.7%
Obstructive sleep apnea	12	28.6%
Chronic obstructive pulmonary disease	9	21.4%
Chronic kidney disease	6	14.3%
Coronary artery disease	5	11.9%

*BMI *body mass index*, SD *standard deviation*. *Categorical variables presented as n (%); continuous variables as mean ± SD or median (range)

Regarding smoking status, the majority of patients were never-smokers (*n* = 24, 57.1%), with 12 (28.6%) being former smokers, and 6 (14.3%) current smokers at the time of repair. The majority of patients were white (*n* = 41, 97.6%). Twelve patients (28.6%) had a history of prior radiation, and one patient (2.4%) was on active chemotherapy at the time of repair. Nineteen patients (45.2%) had a history of active or prior malignancy.

Common comorbidities included hypertension (*n* = 31, 73.8%), hyperlipidemia (*n* = 20, 47.6%), diabetes (*n* = 15, 35.7%), obstructive sleep apnea (*n* = 12, 28.6%), chronic obstructive pulmonary disease (*n* = 9, 21.4%), chronic kidney disease (*n* = 6, 14.3%), and coronary artery disease (*n* = 5, 11.9%).

### Ostomy and hernia characteristics

Ostomy and hernia characteristics are summarized in Table 2. The majority of patients had an end ostomy (*n* = 40, 95.2%), with two patients (4.8%) having a loop ostomy. Stoma distribution included 20 colostomies (47.6%), 12 ileostomies (28.6%), and 9 urostomies (21.4%), with one patient (2.4%) having both colostomy and urostomy. 18 stomas (42.9%) were located in the right lower quadrant, with 16 (38.1%) in the left lower quadrant, 5 (11.9%) in the right upper quadrant, 2 (4.8%) in the left upper quadrant, and one patient (2.4%) with ostomies in both the right and left lower quadrants. The mean time from ostomy creation to hernia repair was 120.6 months (median 83.9 months; range 7.6–529.8 months).

**Table 2:** Ostomy and hernia characteristics (*N* = 42)

Characteristic	*N*	%
*Ostomy type*		
Colostomy	20	47.6%
Ileostomy	12	28.6%
Urostomy	9	21.4%
Colostomy + urostomy	1	2.4%
*Ostomy configuration*		
End ostomy	40	95.2%
Loop ostomy	2	4.8%
*Stoma location*		
Right lower quadrant	18	42.9%
Left lower quadrant	16	38.1%
Right upper quadrant	5	11.9%
Left upper quadrant	2	4.8%
Bilateral (RLQ + LLQ)	1	2.4%
*Time from ostomy creation to repair, mo*		
Mean (range)	120.6 (7.6–529.8)	–
Median	83.9	–
*Prior parastomal hernia repair*	23	54.8%
Primary suture repair	2	–
Sugarbaker repair	11	–
Keyhole repair	10	–
*Hernia defect area, cm*^*2*^		
Median (range)	20 (2–120)	–

*RLQ *right lower quadrant*, LLQ *left lower quadrant,* RUQ *right upper quadrant*, LUQ *left upper quadrant*. *Time reported in months

Twenty-three patients (54.8%) had undergone at least one prior parastomal hernia repair prior to the index operation: 2 primary suture repairs, 11 Sugarbaker repairs, and 10 keyhole repairs. Median hernia defect area at time of repair was 20 cm^2^ (range 2–120 cm^2^).

### Perioperative details

Perioperative details are summarized in Table 3. All patients underwent primary repair utilizing Duramesh mesh suture. The most common operative approach was an open repair (*n* = 28, 66.7%), followed by robotic repair (*n* = 7, 16.7%), hybrid repair (*n* = 4, 9.5%), and laparoscopic repair (*n* = 3, 7.1%). Two patients (3.8%) undergoing robotic repair required conversion to an open procedure. Mean operative time was 225 ± 112 min (range 106–737 min).

**Table 3:** Perioperative details (*N* = 42)

Variable	*N*	%
*Operative approach*		
Open	28	66.7%
Robotic	7	16.7%
Hybrid	4	9.5%
Laparoscopic	3	7.1%
Conversion to open (of robotic)	2	28.6%
Operative time, mean ± SD (range), min	225 ± 112 (106–737)	–

All patients underwent primary repair with Duramesh mesh suture (Mesh Suture Inc., Chicago, IL). Conversion rate calculated among robotic cases only

### Postoperative outcomes

Postoperative outcomes are summarized in Table 4. Mean hospital length of stay was 6.4 ± 6 days (range 2–30 days). Twelve patients (28.6%) experienced at least one postoperative complication. Surgical site occurrences were identified in a subset of these patients, including 3 surgical site infections (7.1%), 3 seromas (7.1%), and 4 hematomas (9.5%), and 1 wound dehiscence (2.4%). Postoperative ileus or small bowel obstruction occurred in 5 patients (11.9%).

**Table 4:** Postoperative outcomes (*N* = 42)

Outcome	*N*	%
Hospital LOS, mean ± SD (range), days	6.4 ± 6 (2–30)	–
Any postoperative complication	12	28.6%
*Surgical site occurrences (SSO)*		
Surgical site infection (SSI)	3	7.1%
Seroma	3	7.1%
Hematoma	4	9.5%
Wound dehiscence	1	2.4%
Postoperative ileus/small bowel obstruction	5	11.9%
*Follow-up, months*		
Mean (range)	7.1 (0.4–20.6)	–
Median	6.7	–
Hernia recurrence	23	54.8%
First time repair (no prior repair)	8	30.8%
Redo repair (prior repair history), p = 0.050	16	61.5%
Time to recurrence, mean (range), months	6.4 (0.1–12.9)	–
Redo repair within follow-up period	7	30.4% of recurrences
Suture pull-through / mesh suture mechanical failure	0	0%

*LOS *length of stay,* SSO *surgical site occurrence*, SSI, *surgical site infection. Redo repair rate calculated among patients with confirmed recurrence. Mean follow-up 7.1 months

At a mean follow-up of 7.1 months (median 6.7 months; range 0.4–20.6 months), 23 patients (54.8%) developed recurrence of their parastomal hernia. When stratified by prior repair history, patients undergoing redo parastomal hernia repair had significantly higher recurrence rates compared to those undergoing first time repair (61.5% vs. 30.8%, *p* = 0.05). Mean time to recurrence was 6.4 months (range 0.1–12.9 months). Among patients who developed recurrence, 7 (30.4%) underwent subsequent redo repair within the follow-up period. In examining the mechanism of failure of the repair during the redo operation, there did not appear to be any cases of suture pull-through or mechanical failure of the mesh suture.

## Discussion

A durable parastomal hernia repair remains one of the most persistent and unsolved challenges in the realm of abdominal wall surgery. Despite new surgical technologies and technical refinement, existing data consistently demonstrate recurrence rates approaching 40% at long-term follow-up, regardless of repair technique. This is still likely underrepresentative of the true recurrence rate given the limited follow-up available in most published series. It is widely accepted that parastomal hernias are a unique problem, as a stoma requires a fascial defect through which functioning bowel must pass, limiting the ability to apply conventional hernia principles. This challenge even extends to parastomal hernia prevention, with multiple randomized control trials demonstrating prophylactic mesh placement at the time of stoma creation does not yield any significant benefit [16, 17]. In addition, the patients with parastomal hernias often suffer from complex medical comorbidities. In our cohort, nearly half of patients had a history of active or prior malignancy, 28.6% had a history of prior radiation, and 54.8% of patients had undergone at least one prior attempted parastomal hernia repair. This latter finding likely contributed to the higher overall recurrence rate observed, as patients with a prior repair history experienced recurrence at twice the rate of those undergoing primary repair (61.5% vs. 30.8%, *p* = 0.05), consistent with the well-established pattern of durability with each successive repair attempt. In this context, the high rates of recurrence are certainly understandable.

We hypothesized that use of Duramesh, a multifilament substrate, may reduce the high rate of suture pull-through and subsequent propagation of the stomal aperture, improving recurrence rates. Unfortunately, we identified an overall recurrence rate of 54.8% at a mean follow-up of 7.1 months, a rate consistent with the broader published literature. The landmark systemic review from Hansson et al. identified an odds ratio of 8.9 for recurrence following primary suture repair compared to mesh repair, and concluded that suture-only repair should be abandoned in favor of mesh reinforcement [10]. Shakarchi and Williams similarly identified a pooled recurrence rate approaching 50% for open suture-based techniques, and the Rendell and Pauli review of parastomal hernia repair notes that primary fascial repair carries recurrence rates as high as 48–66% [11, 18].

Our series suggests that Duramesh, despite its theoretical biomechanical advantages over conventional suture, does not overcome the fundamental limitations of primary repair when used in isolation. Interestingly, albeit in a small sample size, the patients who underwent redo parastomal hernia repairs following initial repair with Duramesh, we did not identify any clear incidents of suture pull-through or fascial disruption. Rather, the permanent, dynamic nature of the remaining stomal trephine likely plays a larger role in repair failure. We suspect the remaining aperture, despite adequate closure, still allows adjacent bowel or other abdominal contents to herniate into the opening despite near-obstructing levels of fascial tightness during initial repair. In our series, a single patient with a history of multiply recurrent parastomal hernia repair, including a prior Sugarbaker repair, was found to have a “loose” stoma aperture within a month of primary repair. She was taken for redo repair with placement of an additional suture to obtain nearly obstructive levels of tightness around the stomal conduit, and despite this she re-recurred during this same hospitalization, and required another operation with addition of mesh. This highlights that although the aperture can be closed clinically to a significant degree, we suspect the overall laxity and malleability of the bowel allows re-herniation even without typical failure of the suture or fascial pull-through. Taken together, our findings suggest that primary parastomal hernia repair with Duramesh alone is not a durable strategy and should be reserved only for circumstances in which more definitive mesh-based repair is not feasible. As with traditional suture-based primary repair, it would be appropriate to counsel patients on the high likelihood of recurrence, even at short-interval follow-up.

This study has several important limitations. First, as a single-center retrospective series, it is subject to selection bias in repair technique assignment. Second, the mean follow-up of 7.1 months is short relative to the known natural history of parastomal hernia recurrence, and thus the recurrence rates are likely to increase with longer surveillance. Our practice changed immediately as we noted early recurrences and we sought to describe our experience as a cautionary one for others considering this as a viable primary repair method. Finally, the heterogeneity of ostomy type and location as well as the mixture of concomitant procedures performed at the time of repair, including bowel resections, ostomy re-sitings, and additional hernia repairs, reflects the complexity of this patient population but limits the ability to isolate the effect of the repair technique.

Overall, primary parastomal hernia repair utilizing Duramesh suture alone is associated with high recurrence rates and should not be considered a durable standalone repair strategy. Our results affirm the longstanding message in the parastomal hernia literature that suture-only repair, regardless of the suture construct employed, remains insufficient as a definitive repair strategy. Without mesh reinforcement, the stomal aperture is an unavoidable defect through which hernias will certainly recur. Further prospective study with longer follow-up and larger sample sizes, specifically with Duramesh fascial closure with existing Sugarbaker or keyhole techniques is necessary to define whether or not mesh suture may have a role in a more durable parastomal hernia repair.
